# Dimethylsulfoxide excerbates cisplatin-induced cytotoxicity in Ehrlich ascites carcinoma cells

**DOI:** 10.1186/s12935-015-0258-1

**Published:** 2015-10-29

**Authors:** Abdel-Moneim M. Osman, Ali A. Alqahtani, Zoheir A. Damanhouri, Sameer E. Al-Harthy, Mohamed F. ElShal, Wafaa S. Ramadan, Fatemah Kamel, Mohamed A. M. Osman, Lateef M. Khan

**Affiliations:** Pharmacology Department, Faculty of Medicine, King Abdulaziz University, Jeddah, Saudi Arabia; Pharmacology Unit, National Cancer Institute, Cairo University, Giza, Egypt; Department of Biochemistry, Faculty of Science, King Abdulaziz University, Jeddah, Saudi Arabia; Molecular Biology Department, Genetic Engineering and Biotechniology Department, Minoufia University, Minoufia, Egypt; Anatomy Department, Faculty of Medicine, King Abdul Aziz University, Jeddah, Saudi Arabia; College of Dentistry, Russian University, Cairo, Egypt

**Keywords:** Cisplatin, Dimethylsulfoxide, Cytotoxicity

## Abstract

**Background:**

Cisplatin (CIS) is a potent antineoplastic agent with high therapeutic efficacy against many kinds of tumors. Its use is limited by its nephrotoxicity. The aim of this work was to minimize cisplatin effective dose and the possible reduction of its severe side effects. The present study was designed to assess the role of sulfur containing agent dimethyl sulfoxide (DMSO) on sensitization of mammary carcinoma, Ehrlich ascites carcinoma (EAC), to the action of cisplatin and at the same time the possible protective effect against cisplatin induced nephrotoxicity in experimental animals.

**Methods:**

To evaluate these effects we have explored the cisplatin effect on the survival time of tumor-bearing animals, tumor weight, cisplatin cellular uptake, apoptosis induction and cell cycle distribution and renal function in presence and absence of DMSO.

**Results:**

Cisplatin at dose of 4.5 mg/kg increased the mean survival time of tumor bearing mice to 37 days compared with tumor bearing control mice. Pretreatment of tumor bearing mice with DMSO 50 % (2 ml/kg equal to 1 gm/kg) 2 h. before cisplatin showed a significant increase in their mean survival time 43 days compared to cisplatin treated animals. DMSO pretreatment retained rat’s serum urea and creatinine levels to normal compared to animals treated with cisplatin alone.

**Conclusion:**

DMSO pretreatment enhanced the cytotoxic activity of cisplatin against the growth of EAC in vivo and showed protective effects against cisplatin-induce nephrotoxicity.

## Background

Cisplatin is one of the most active cytotoxic agents in clinical use that has proven efficacy against numerous human solid malignancies such as bladder, cervical, head and neck, esophageal, and small cell lung cancer [1]. However, some tumors such as colorectal and non-small cell lung cancers have intrinsic resistance to cisplatin, while others such as ovarian or small cell lung cancers develop acquired resistance after the initial treatment [2]. Recently, the cisplatin clinical usefulness is limited by chemoresistance and its side effects such as ototoxicity and nephrotoxicity [3]. Chemosensitization is one strategy to overcome chemoresistance. It is based on the use of one drug or natural products to enhance the efficacy of antineoplastic drugs by modulating one or more mechanisms of resistance. DMSO is an organo-sulfur compound with a reactive oxygen species scavenger [4]. Dimethyl sulfoxide has many chemical properties make it suitable as pharmaceutical carrier for many drugs, electrolytes and other molecules [5–7]. Uribe et al. [8], found that DMSO treatment potentiated the effect of cisplatin and killed more sensory hair cells than treatment with cisplatin alone. They interpreted their results as DMSO could enhance cisplatin cytotoxicity by facilitating cisplatin entry into cells, increasing its intracellular concentration and likelihood of binding to DNA. In light of these findings the goal of this study is to examine the possible effect of DMSO pretreatment in enhancing the antitumor activity of CIS by examining CIS antitumor activity, apoptosis induction, cell cycle distribution and cisplatin cellular uptake into tumor cells. In addition, examining the possible renal protective effect of DMSO against CIS triggered nephrotoxicity.

## Methods

### Drugs and chemicals

Cisplatin (CIS) and dimethylsulfoxide (DMSO) were purchased from Sigma Aldrich Co. (Saint Louis, Missouri, USA). The stock solution of both drugs (dissolved in phosphate buffer saline (PBS) and preserved at −20 °C. The solutions were diluted in normal saline immediately before each experiment to the desired final concentration.

### Animals and tumor

Female Swiss albino mice (8 weeks of age, 20–22 g body weight) and Female Wistar albino rats (8–10 weeks of age, 180–200 g body weight) were obtained from King Fahd Medical Research center, King Abdulaziz University, Jeddah, Saudi Arabia. The animals were acclimatized for 1 week before each experiment. A commercial balanced diet and water ad libitum were provided throughout the experiment. The Ehrlich ascites carcinoma cells (EAC) cells was obtained through the courtesy of National cancer institute (Cairo, Egypt) and maintained in our laboratory by weekly I.P. transplantation of 2.5 × 10^6^ cells/mouse. This study was approved by the Institutional ethical committee of King Abdulaziz hospital.

### Evaluation of antitumor activity

The effect of DMSO on the antitumor activity of CIS against the growth of EAC was evaluated using the modified regimen of Donenko et al. [9]. Ehrlich ascites carcinoma cells were inoculated i.p. into forty swiss albino mice (20–22 g) 2.5 × 10^6^ cells/mouse. Twenty-four hours later, mice were equally divided into four groups. Group I injected with normal saline i.p. (0.2 ml/20 gm) and served as control group. Group II administered with CIS (4.5 mg/kg i.p.) while group III received a single dose of DMSO (50 %, 2 ml/kg i.p.). Group IV received DMSO followed by cisplatin (4.5 mg/kg i.p.) 2 h later. Average survival days of mice and long term survivors are defined as the mice survived to the end of the experiment (45 days) with no apparent tumor.

### Assessment of tumor weight

Ehrlich ascites carcinoma cells were collected from the ascitic fluid of female Swiss albino bearing mice 8–10 days old ascites tumor. 1 × 10^6^ EAC cells were injected intramuscularly in right thigh of female Swiss albino mice selected for the experiment on day 0. The next day, animals were randomized and divided into four groups each treatment group contains 10 animals. Treatment with DMSO and/or Cisplatin was proceed as the above paragraph.

On day 18, tumor bearing thigh of each animal was shaved and longest and shortest diameters of the tumor were measured with the help of Vernier Caliper. Tumor weight of each animal was calculated using the following formula:

$${\text{Tumor weight }}\left( {\text{mg}} \right){ = 0} . 5 {\text{ (Length }}\left( {\text{mm}} \right) \times \left( {\text{width mm}} \right)^{ 2}$$

The percent tumor growth inhibition was calculated on day 18 by comparing the average values of treated groups with that of tumor bearing control group.

### Measurement of cisplatin cellular uptake

EAC were inoculated i.p. into 20 Swiss albino mice (20–22 g) 10 × 10^6^ cells/mouse. Twenty-four hours later mice were divided into two groups (10 mice each). Group I animals were treated with cisplatin (4.5 mg/kg, i.p.). Group II animals were treated with 50 % DMSO (2 ml/kg, i.p.) followed by cisplatin injection (4.5 mg/kg, i.p) 2 h. Later. Animals were sacrificed by cervical dislocation 24 h. after treatment then the tumor cells were withdrawn and washed twice with PBS then the cells were counted. For drug uptake analysis, cells (1 × 10^6^) were suspended in 1 % HNO_3_ for 24 h. at 70 °C to be digested. Lysed cells were analyzed by ICP-MS (Thermo scientific, iCAP 6000 series; USA). Provides a quantitative analysis of the concentration of an element in aqueous solution [10].

### Assay of apoptosis

Apoptosis cells were quantified by annexin V-FITC-propodium iodide double staining, using an annexin V-FITC apoptosis detection kit. EAC were inoculated i.p. into 40 Swiss albino mice (20–22 g) 10 × 10^6^ cells/mouse. Twenty-four hours later mice were divided into four groups (10 mice each). Group I animals were treated with normal saline i.p. (2 ml/kg, i.p.) and served as control group. Group II animals were treated with 50 % DMSO (2 ml/kg, i.p.). Group III animals were received cisplatin (4.5 mg/kg, i.p.). Group IV animals were treated with 50 % DMSO (2 ml/kg, i.p.) followed by cisplatin (4.5 mg/kg, i.p.) 2 h later.

Animals were sacrificed by cervical dislocation 24 h after treatment then the tumor cells were withdrawn and washed twice with PBS and resuspended in 100 µl annexin V incubation reagent prepared by mixing (binding buffer 10×, PI, annexin V-FITC and deionized water) for each sample. The solution was incubated in the dark for 15 min at RT. Then 400 μl 1× of binding buffer were added to each sample and process by flow cytometry (NAVIOS Beckman Coulter, USA) within 1 h for maximal signal.

### Cell cycle analysis

Ehrlich ascites carcinoma cells were inoculated i.p. into sixty swiss albino mice (20–22 g) 10 × 10^6^ cells/mouse and processed as mentioned in the paragraph of Apoptosis. Tumor cells were obtained 24 h. after cisplatin treatment. Cell cycle analysis was performed using flow cytometer (Becto Dicknson, BD, FACScalbur, USA according to the method of Pozarowiski P., Darzynkiewicz [11].

### Effect of CIS and/or DMSO on kidney function and renal histopathology

Twenty male wister rat were divided into four equal groups, 5 animals each. Group I animals were received normal saline i.p. (2 ml/kg, i.p.) and reserved as control group. Group II animals were treated with 50 % DMSO (2 ml/kg i.p.). Group III animals were treated with cisplatin (7.5 mg/kg, i.p.). Finally, Group IV animals were treated with 50 % DMSO (2 ml/kg, i.p.) followed by cisplatin (7.5 mg/kg, i.p.) 2 h later. At the end of the experiment period (72 h), rats were anesthetized and blood samples were collected from the ophthalmic artery in the orbital rim and rapidly centrifuged for serum separation that was stored at −80 °C to evaluate serum urea and creatinine levels.

The dissected rat kidney was cut into small pieces and immersed immediately in 10 % neutral buffered formalin, for light microscope study.

### Evaluation of serum creatinine concentration

The serum creatinine concentration was estimated by alkaline picrate method using the commercially available kit [12].

### Evaluation of serum urea concentration

The blood urea was estimated by Berthelot method [13] using the commercially available kit.

### Statistical analysis

Statistical analysis was performed using SPSS (Statistical package of social science, version 16).One way analysis of variance (ANOVA) followed by least significant difference (LSD) for post hoc analysis was used for multiple comparisons. Statistical significance was acceptable to a level of p ≤0.05.

## Results

### Survival of tumor bearing mice

Table 1 shows the survival of Erlich ascites carcinoma bearing mice after treatment with cisplatin and/or DMSO. Tumor-bearing control mice showed a mean survival time of 17 days, whereas,administration of a single dose of cisplatin (4.5 mg/kg, i.p.) increased the mean survival time to 37 days, with 50 % long term survivors. Treatment with 50 % DMSO 2 h. before increased the mean survival time of tumor-bearing mice to 43 days with 80 % survivors.

**Table 1:** Effects of cisplatin and/or DMSO on the survival time of mice bearing EAC cells

Groups	Mean survival time (days)	Long term survivor %
Normal saline (2 ml/kg)	17 ± 0.45	0
50 % DMSO (2 ml/kg)	18 ± 0.37	0
Cisplatin (4.5 mg/kg)	37 ± 3.41^a^	50
Cisplatin + 50 % DMSO (4.5 mg/kg + 2 ml/kg)	43 ± 2.10^a,b^	80

Data represent the mean ± SD of ten mice

^a^Significantly different from control at *P* value < 0.05)

^b^Significantly different from cisplatin at P value <0.05, one way ANOVA with LSD post test)

### Assessment of tumor weight

Table 2 shows Anti-tumor efficacy of cisplatin and/or DMSO which was expressed as the percentage of tumor growth inhibition calculated on day 18 by comparing the average tumor weight of treated groups with that of tumor bearing control group. Tumor growth in saline treated animals was taken to be 100 %. Cisplatin treatment (4.5 mg/kg, i.p.) showed 61 % inhibition of tumor growth. Pretreatment with DMSO 50 % (2 ml/kg, i.p.) showed 80 % inhibition of growth of solid EAC.

**Table 2:** Average weights and tumor inhibition of ehrlich solid tumor 18 days after treatment

Treatment groups	Avg. tumor weights (mg)	Tumor growth inhibition %
Normal saline	910 ± 22.8	0
50 % DMSO	761 ± 17.1	16
Cisplatin (4.5 mg/kg)	352 ± 8.4^a^	61
50 % DMSO + Cisplatin (4.5 mg/kg)	180 ± 5.8^a,b^	80

Ehrlich ascites carcinoma cells were injected intramuscularly in right thigh of female Swiss albino mice The next day, animals were randomized and divided into four groups each treatment group contains 10 animals. Group I injected with normal saline (2 ml/kg, i.p.) and served as control group. Group II treated with 50 % DMSO (2 ml/kg, i.p.). Group III treated with cisplatin (4.5 mg/kg, i.p.). Group IV treated with (2 ml/kg, i.p.) of 50 % DMSO followed by cisplatin (4.5 mg/kg, i.p.) 2 h later. Each data represent the mean ± SEM

^a^Significantly different from normal saline treated group at P value <0.05

^b^Significantly different from cisplatin treated group at P value <0.05

### Effect of cisplatin and/or DMSO on apoptosis induction in EAC cells

**Fig. 1:**
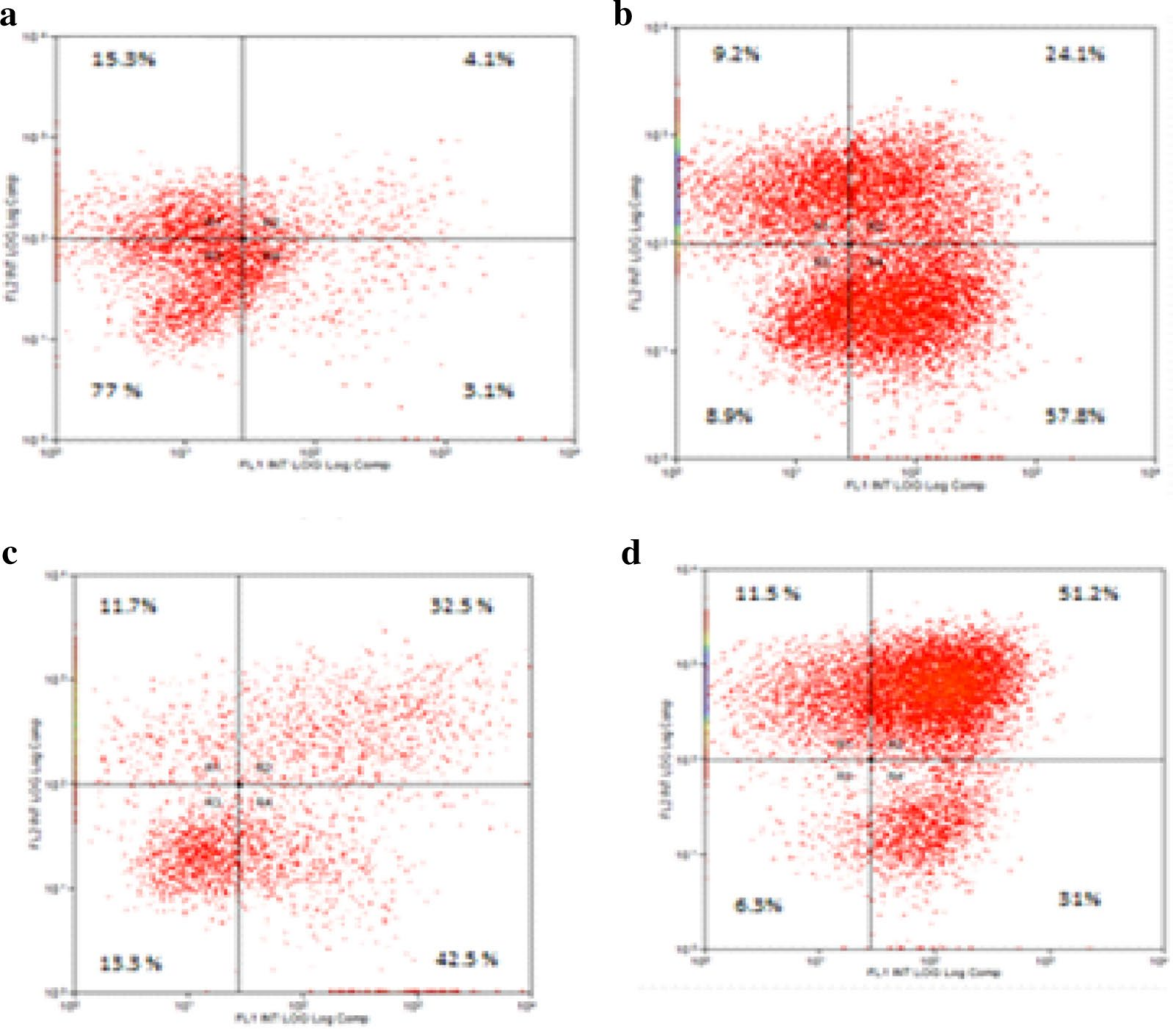
Effect of cisplatin and/or DMSO on apoptosis induction in EAC cells withdrawn 24 h. after treatment. Apoptosis was analyzed by staining with propiduim iodide (PI, *y-axis*) and annexin—FITC (*x-axis*). **a** EAC cells withdrawn from normal saline (2 ml/kg, i.p.) treated animals. **b** EAC cells withdrawn from animals treated with 50 % DMSO (2 ml/kg, i.p.). **c** EAC cells withdrawn from animals treated with cispalin (4.5 mg/kg, i.p.) alone. **d** EAC cells withdrawn from animals pretreated with 50 % DMSO (2 ml/kg, i.p.) followed by cispatin (4.5 mg/kg, i.p.). The percentage of cells in each quadrant is indicated (*R1* necrosis, *R2* late apoptosis, *R3* live Cells, *R4* early apoptosis). The experiment was repeated twice each one in duplicate

The percentage of early apoptotic cells (Annexin V-positive cells) were dramatically increased after treatment with cisplatin in comparison to the control cells (% early apoptosis cells). Also, pretreatment with 50 % DMSO (2 ml/kg, i.p.) followed by cisplatin (4.5 mg/kg, i.p.) increased the percentage of early apoptotic cells (Annexin V-positive cells) significantly compared with EAC cells withdrawn from animals treated with cisplatin (4.5 mg/kg, i.p.) alone Fig. 1.

### Effect of CIS and/or DMSO treatment on cell cycle phase progression in ehrlich cells

**Fig. 2:**
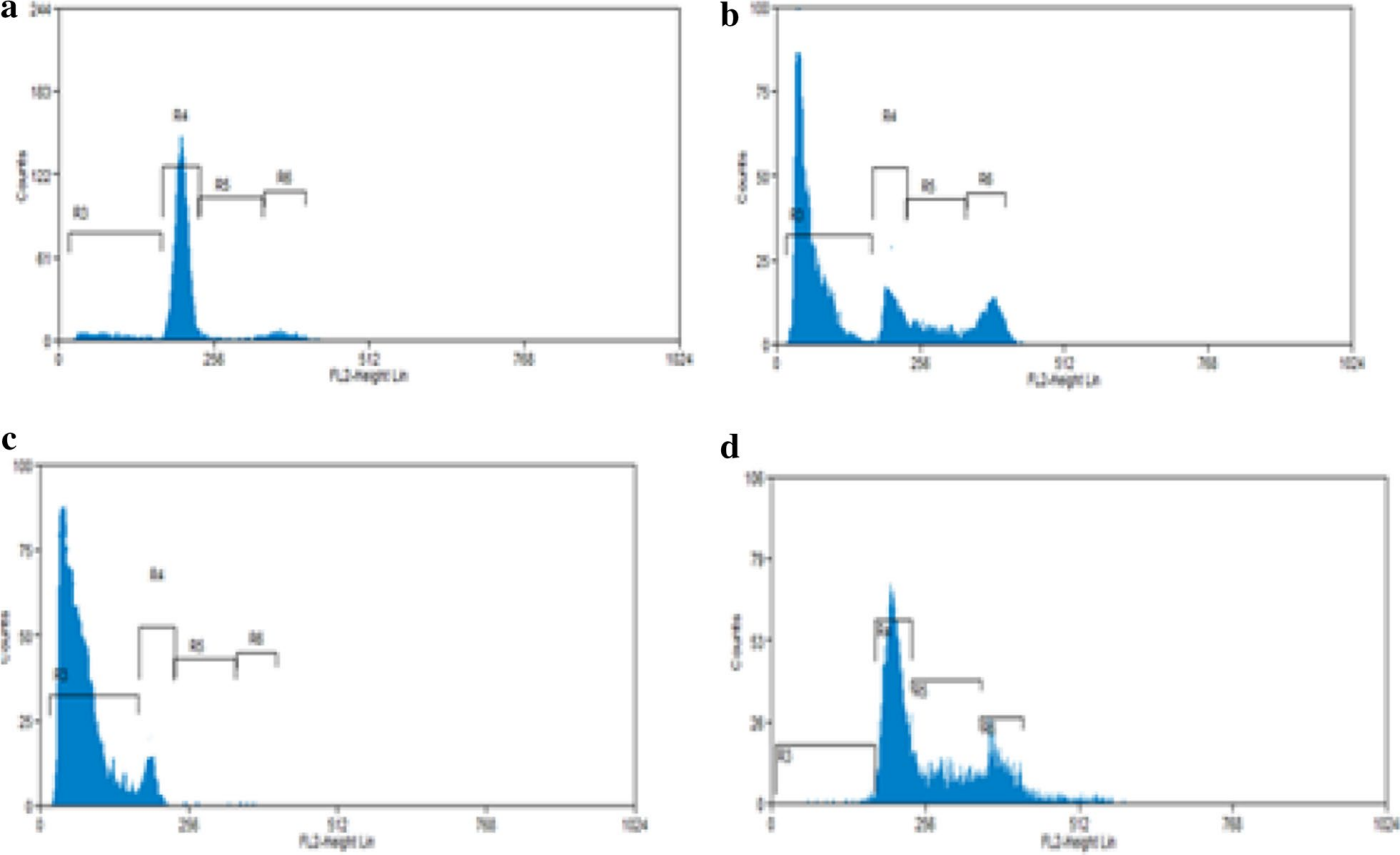
Effect of cisplatin and/or DMSO on cell cycle phase distribution of EAC cells after 24 h of treatment. Cell cycle distribution was analyzed by staining with propidium iodide (PI). **a** Cells that withdrawn from animals treated with normal saline (2 ml/kg, i.p.), **b** cells that withdrawn from animals treated with cisplatin (4.5 mg/kg, i.p.). **c** Cells that withdrawn from animals pretreated with 50 % DMSO (2 ml/kg, i.p.) followed by Cisplatin (4.5 mg/kg, i.p.). **d** Cells withdrawn from animals treated with 50 % DMSO (2 ml/kg, i.p.). The experiment was repeated twice

Figure 2 shows the percent distribution of G_0_/G_1_, S, and G_2_/M of tumor cells 24 h after treatment with cisplatin and/or DMSO. Cisplatin treatment (4.5 mg/kg, i.p.) accumulated the cells in G_0_/G_1_ phase by 42 %. Pretreatment with 50 % DMSO (2 ml/kg, i.p.) followed by cisplatin (4.5 mg/kg, i.p.) significantly increase the accumulation in G_0_/G_1_ phase by 57 %.

### Cisplatin cellular uptake in Ehrlich ascites cells

Table 3 shows the cellular level of CIS in Ehrlich ascites cells after a single dose of cisplatin (4.5 mg/kg, i.p.) and/or DMSO 2 h before (50 %, 2 ml/kg, i.p.). All the time point tested showed that DMSO pretreatment increased the cellular uptake of CIS in the tumor cells (Table 3).

**Table 3:** Effect of DMSO pretreatment on the cellular uptake of cisplatin in EAC cells using ICP-MS

Cisplatin concentration (ng/10^6^ cells)			
	6 h after treatment	24 h after treatment	48 h after treatment
Cisplatin (4.5 mg/kg)	7 ± 0.26	14 ± 0.47	18 ± 0.58
Cisplatin (4.5 mg/kg) and 50 % DMSO (2 ml/kg)	15 ± 0.23 ^a^	28 ± 0.45^a^	35 ± 1.25^a^

Cisplatin was injected (4.5 mg/kg, i.p.) in tumor-bearing mice pretreated with DMSO (2 ml/kg, i.p.) or saline. After 6, 24 and 48 h of treatment, cells were withdrawn and washed twice with PBS, counted then digested by using 1 % nitric acid. Data are expressed as mean ± S.E.M (n = 10)

^a^Significantly different from corresponding CIS at P value <0.05

### Effects of DMSO on cisplatin induced-nephrotoxicity

Table 4 represents the kidney function (serum creatinine and urea) of treated animals. DMSO treatment had no significant effect on urea and serum creatinine levels compared with control group. Serum urea and creatinine average levels were significantly elevated in cisplatin treated animals by about 237 and 3 mg/dl, respectively. While DMSO pretreatment of cisplatin treated animals return both urea and creatinine levels nearly to normal values (73 and 0.73 mg/dl, respectively).

**Table 4:** Effect of treatment with cisplatin and/or DMSO on serum urea and creatinine levels

Treated groups	Parameters	
	Urea (mg/dl)	Creatinine (mg/dl)
Normal saline	48 ± 1.7	0.51 ± 0.02
50 % DMSO	52 ± 0.49	0.54 ± 0.04
Cisplatin	237 ± 11.51^a^	3 ± 0.25^a^
Cisplatin and 50 % DMSO	73 ± 3.44^a,b^	0.73 ± 0.11^a,b^

Values are expressed in mean ± SEM (n = 5)

^a^Significantly different from control at P value <0.05

^b^Significantly different from CIS at P value <0.05, one way ANOVA with LSD post test

**Fig. 3:**
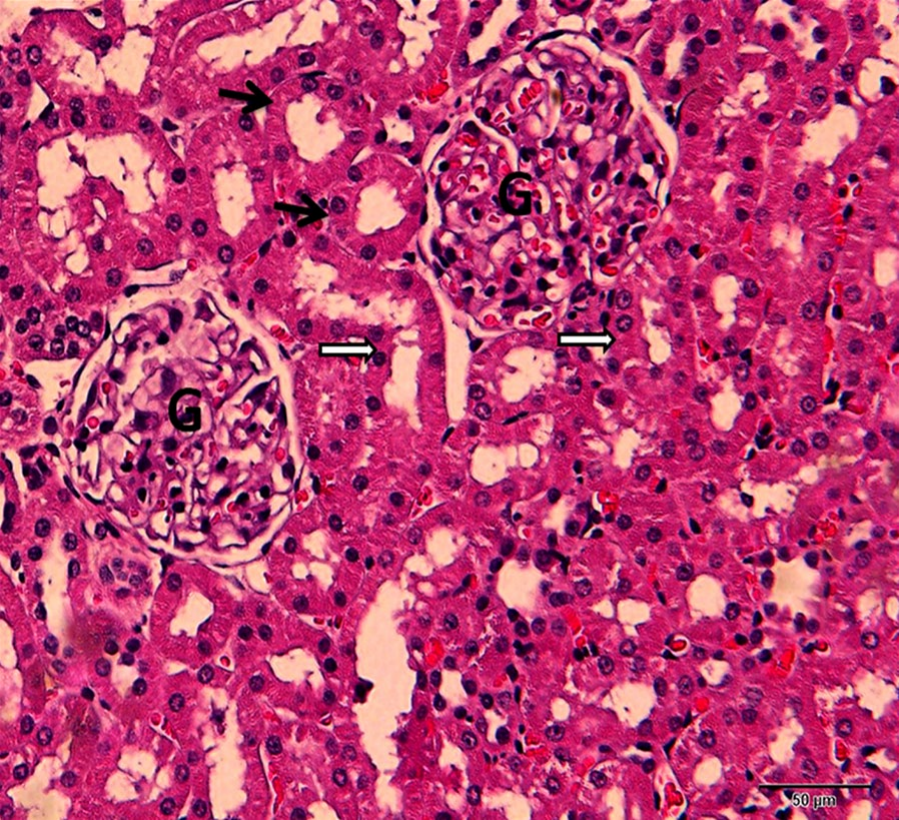
A photomicrograph of a section of rat kidney from control group treated with normal saline (2 ml/kg, i.p.) showing proximal convoluted tubules (*black arrows*) having cuboidal cells with eosinophilic cytoplasm and round nuclei situated in the center or near the base of the cells. The distal convoluted tubules (*white arrows*) also reveal eosinophilic cuboidal cells with round nuclei. Glomeruli: G (H&E ×400)

**Fig. 4:**
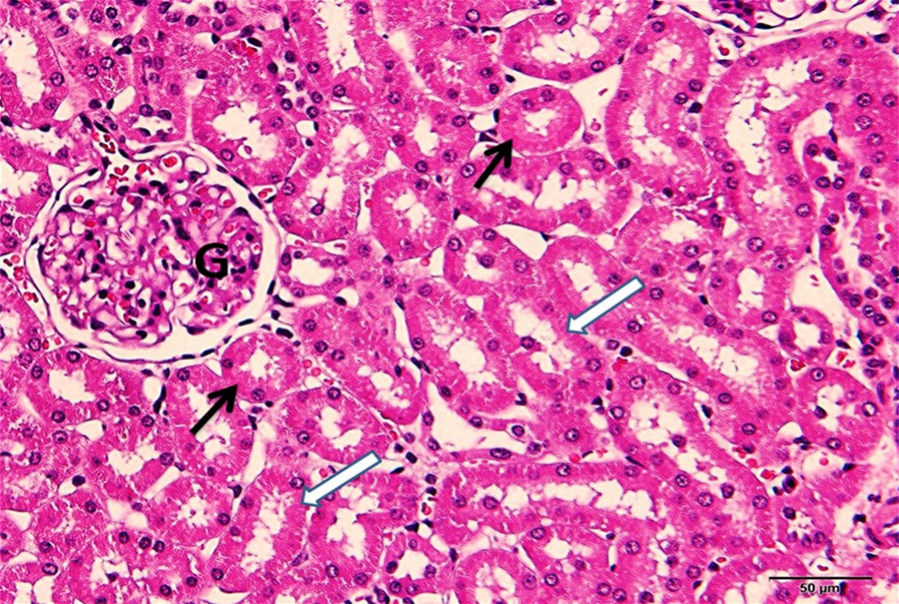
A photomicrograph of a section of rat kidney treated with 50 % DMSO (2 ml/kg, i.p.) showing well organized architecture. The epithelium of most of the proximal (*black arrows*) and distal convoluted tubules (*white arrows*) show normal structure. Glomerulu: G. (H&E ×400)

**Fig. 5:**
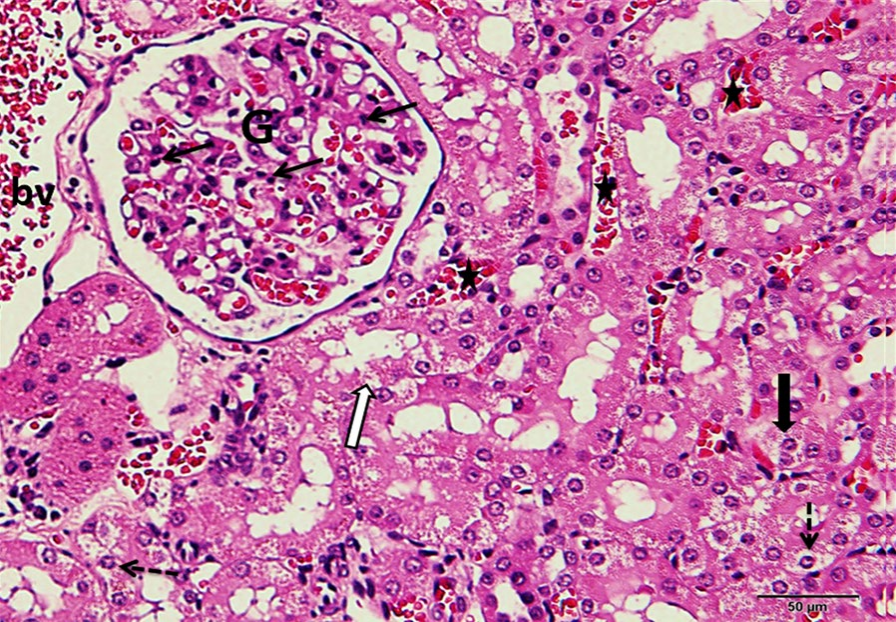
A photomicrograph of a section of rat kidney treated with cisplatin (7.5 mg/kg, i.p.) showing disruption of epithelium of the proximal convoluted tubule (*thick black arrow*) and distal convoluted tubule (*white arrow*). Karyorrhetic nuclei within the vacuolated cytoplasm of the tubular epithelium (*dashed arrows*) are observed. (*filled star*) Pyknotic nuclei are noticed (*thin black arrows*) in the endothelial and mesangial cells of the glomerulus (G). Dilated congested blood vessels (bv) and intertubular capillaries are also noticed (H&E ×400)

**Fig. 6:**
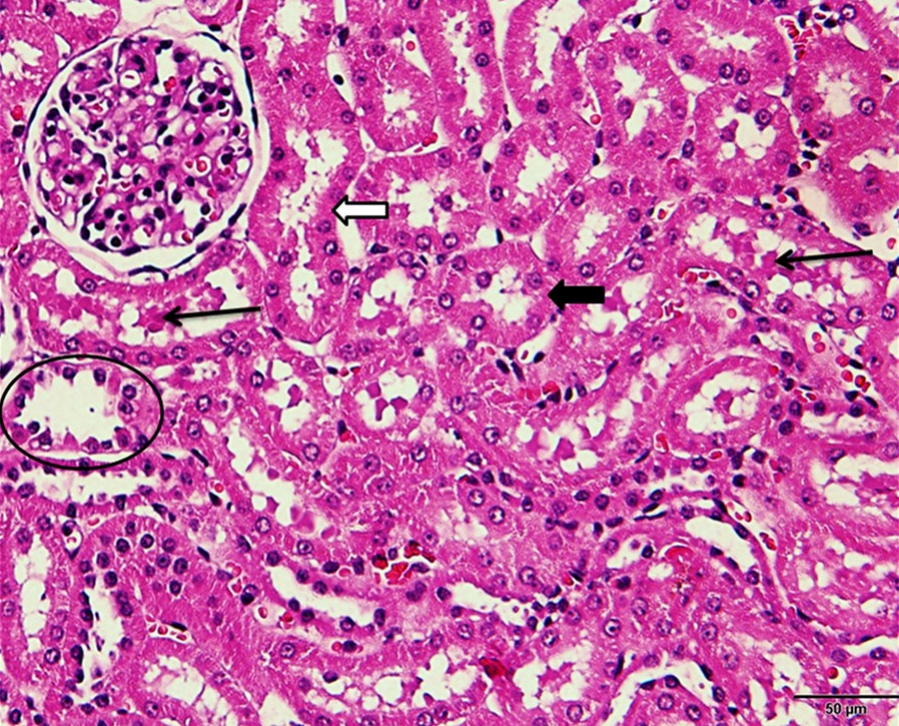
A photomicrograph of a section of rat kidney treated with cisplatin (7.5 mg/kg, i.p.) after 2 h of pretreatment with 50 % DMSO (2 ml/kg, i.p.) showing most of the proximal convoluted tubules (*thick black arrow*) and distal convoluted tubules (*white arrow*) revealing normal tubular epithelium. Few reveal attenuated and desquamated epithelium (*white circle*). Others contain intra-tubular pink homogenous casts (*thin black arrows*) (H&E ×400)

Figures (3, 4) showed normal kidney tissue with no abnormalities when the animals treated with normal saline or DMSO, respectively. While treatment with cisplatin (7.5 mg/kg, i.p.), there were marked necrosis in proximal tubules and degeneration of the tubular epithelial cells (Fig. 5). Pretreatment with 50 % DMSO(2 ml/kg, i.p.), decreased the cisplatin induced tubular necrosis (Fig. 6) comparing with cisplatin treated animals.

## Discussion

Cisplatin is the most widely used cytotoxic drug in the treatment of many kinds of tumors either alone or in combination with other cytocidal agents. However, its clinical uses are limited by its detrimental adverse effects including nephrotoxicity. Chemosensitization is one strategy that may be used to decrease the anti-tumor dose and toxicity. A variety of approaches have been tried to enhance the cytotoxic effects of chemotherapeutic agents and at the same time decreased their toxicity. Among the potential chemosensitizer is dimethyl sulfoxide which has chemopreventive [14] and cytotoxic activity [15]. This study focused on investigating whether DMSO would enhance the cisplatin cytotoxic effects against the growth of EAC cells in vivo and the possible protective effect against cisplatin induced nephrotoxicity. The possible modulatory mechanisms were also explored by studying the changes of apoptosis induction, cell cycle phase distribution and cisplatin cellular uptake after treatment with cisplatin in the presence and absence of DMSO. Preliminary studies with different DMSO doses showed that a dose level of 2 ml/kg (2 gm/kg) was of great efficacy and little organ toxicity.

The current study showed that the pretreatment of tumor bearing mice with 50 % DMSO (2 ml/kg, i.p.), significantly enhances the cytotoxic activity of cisplatin against the growth of EAC cells by 1.6-fold increase in the long term survivor compared with animals treated with cisplatin alone (Table 1).

It is well known that cisplatin induced formation of intra and inter-DNA strand cross linkage lead to severe local distortion in the DNA double helical structure lead to cell death [16–18]. For more conformation, the study showed that the treatment of solid Ehrlich tumor bearing mice with 50 % DMSO before cisplatin treatment increased the percentage of inhibition of solid tumor growth to 80 % compared with 61 % in cisplatin treated animals (Table 2).

The above mentioned results have been confirmed by the observed increase in cisplatin cellular uptake after DMSO treatment. Pretreatment with DMSO lead to almost twofolds increase in the cisplatin accumulation ratios in EAC cells compared with corresponding cells treated with cisplatin alone (Table 3). It has been reported that DMSO induces membrane thinning, increases the fluidity of the membrane hydrophobic core and induces transient water pores into the membrane which may facilitate the uptake of cisplatin into tumor cells [5, 19, 20]. This may lead to more cisplatin uptake and consequently lead to cell death. The current study agree with that reported by Uribe et al. [8], who found that DMSO treatment potentiated the effect of cisplatin and killed more sensory hair cells than treatment with cisplatin alone. They also interpreted their results as DMSO could enhance cisplatin cytotoxicity by facilitating cisplatin entry into cells, increasing its intracellular concentration and likelihood of binding to DNA. This finding has been observed in our study where DMSO treatment increased cellular uptake and cytotoxicity of cisplatin against the growth of tumor cells. DMSO may bind to cisplatin after tumor cell entry, where cisplatin-DMSO adducts have greater affinity for DNA, potentiating cisplatin cytotoxicity [21]. Also, our results supports Pommier et al. [22] who reported that DMSO could sensitize cancer cells to the apoptosis or growth arrest and synergistically increases the cytotoxicity of antineoplastic agents against five different human tumor reference cell lines. Moreover, our results showed a significant increase in percentages of early apoptosis in the EAC cells treated with cisplatin and DMSO, compared with cells treated with cisplatin alone (Fig. 1).

In contrary to our previous results, Hall et al. [23] showed inhibition of cytotoxicity and ability to initiate cell death when cisplatin dissolved in DMSO. This discrepancy could be refuted as their study depended on formation of new chemical compound between cisplatin and DMSO, while the principle of our study depends on pretreatment of animals with DMSO before cisplatin. This preempt administration allowed DMSO to distribute into EAC cells and to exerts its possible modulation effect on different targets such as cell membrane and mitochondrial membranes, apoptotic signaling proteins and cell cycle regulators. The current results showed that the pretreatment of the tumor cells withdrawn from animals treated with DMSO before cisplatin, showed a significant increase in the arrested cells in G_0_ compared with cells treated with cisplatin alone (Fig. 2). This could be due to the capability of DMSO to modulate several cell signaling molecules, including cell survival proteins, drug transporters and cell proliferative proteins and its ability to interfere with the expression of anti-apoptotic signals [15, 24]. It is well known that DNA damage caused by different cytotoxic agents, induced cell cycle arrest at G_1_, S, G_2_, thereby preventing replication of damaged DNA or aberrant mitosis which if not repaired, may result in either tumorigenesis or apoptosis [15]. Our results suggested that DMSO induce apoptosis dominantly in a wide variety of tumor cells through targeting many of the cisplatin apoptotic protein as overexpression of P53 [25], P21 [26], Bcl-2 and Bcl2/bax ratio [27] This will able DMSO to potentiate the cisplatin induced-apoptosis and lead to more killing effect. In animals studies the acute nephrotoxicity induced by cisplatin was associated with high level of serum creatinine and blood urea nitrogen [28].

In the current study, rats treated with cisplatin alone showed a significant increase in the levels of serum creatinine and blood urea levels, while in the DMSO pretreated animals the levels nearly return to normal (Table 4).

Previous results by Ali and Mousa, [28] have also reported that DMSO was effective in completely preventing the development of signs of nephrotoxicity of nephrotoxic drug gentamycin (50 mg/kg), in treated rats. In harmonization with our results they stated that treatment with DMSO alone did not alter significantly any of the renal function tests studied. Our biochemical results have been confirmed by histopathological studies of the kidneys of cisplatin treated rats in presence and absence of DMSO. Histopatholgical evaluation in this study showed that cisplatin treatment causes a marked necrosis in proximal tubules and degeneration of the tubular epithelial cells, while pre-treatment with DMSO minimized these histopathological deteriorations (Figs. 5, 6). Moreover, electron microscopic investigation of rat’s kidneys tissues after cisplatin treatment in presence and absence of DMSO confirmed the histopathological findings (data not shown). These results are in a good agreement with Jones et al. [12, 14] and Santos et al. [29] who reported the protective effect of DMSO against cisplatin-induced kidney tissues damage. They referred this protective effect to antioxidant properties of DMSO which result in reserving glutathione and consequently introduce a perfect nephroprotection.

In conclusion, it seems that dimethyl sulfoxide could potentiate the cytotoxic activity of cisplatin by many different molecular mechanisms which need to be carefully investigated to know the exact mechanisms of synergistic interaction between DMSO and cispaltin. Also DMSO could be a proper agent to be applied clinically in many other situations with a dose up to one gram per kilogram body weight.
